# Perioperative Antimicrobial Prophylaxis: Knowledge, Beliefs, and Practices Among Plastic Surgeons in Upper Limb Surgeries

**DOI:** 10.7759/cureus.83732

**Published:** 2025-05-08

**Authors:** Fahad K. Aljindan, Fahad Alhumaid, Ibrahim R Halawani, Sultan K AlMubarak, Jawahir O Altamimi, Noor Al-lababidi

**Affiliations:** 1 Department of Plastic Surgery, King Abdullah Medical City, Makkah, SAU; 2 Department of Plastic Surgery, King Faisal Specialist Hospital and Research Centre, Jeddah, SAU; 3 Faculty of Medicine, King Abdulaziz University, Jeddah, SAU; 4 Plastic and Reconstructive Surgery Department, National Guard Hospital, King Abdulaziz Medical City, Jeddah, SAU; 5 Plastic Surgery Department, King Abdulaziz Medical City, Jeddah, SAU; 6 Plastic Surgery, College of Medicine, King Fahd Hospital of the University, Imam Abdulrahman Bin Faisal University, Dammam, SAU

**Keywords:** knowledge, perioperative antibiotic prophylaxis, plastic surgeons, practice, upper limb surgery

## Abstract

Background: Surgical antimicrobial prophylaxis (SAP) is vital for preventing surgical site infections (SSIs). However, variability in SAP practices and the inappropriate use of antibiotics pose significant challenges in upper limb surgery. Despite its importance, no unified guidelines exist to standardize SAP practices. This study aimed to evaluate the knowledge, beliefs, and practices of plastic surgeons regarding SAP in upper limb and hand surgeries.

Methods: This cross-sectional study was conducted using an online self-administered survey distributed to all Saudi plastic surgeons in March 2023. The questionnaire comprised 13 items assessing demographic characteristics, SAP practices in patients with clean and contaminated surgeries, and awareness of existing guidelines. Analyses were performed using IBM Corp. Released 2017. IBM SPSS Statistics for Windows, Version 25.0. Armonk, NY: IBM Corp.

Results: A total of 65 plastic surgeons were included in the study. The majority (n = 58, 89.2%) were employed in the government sector, whereas 35.4% (n = 23) had 10-15 years of work experience. Most participants administered antibiotics preoperatively for clean (n = 56, 86.2%) and contaminated (n = 63, 96.9%) hand and upper limb surgeries, with higher administration rates observed in contaminated cases. Cefazolin was the most frequently used antibiotic preoperatively (n = 49, 75.4%), while amoxicillin-clavulanate was the preferred postoperative antibiotic for both clean (n = 31, 47.7%) and contaminated (n = 36, 55.4%) hand surgeries. Although 61.5% (n = 40) of the surgeons were aware of SAP guidelines, 30.8% (n = 20) cited a lack of consensus as the key factor influencing their decisions. No significant differences were observed between workplace environments and SAP practices (all χ² tests, p > 0.05).

Conclusion: The findings indicate substantial variability in SAP practices among Saudi plastic surgeons, highlighting the need for clear, evidence-based guidelines to optimize patient care.

## Introduction

Approximately 300,000 surgical site infections (SSIs) occur annually in the United States, resulting in significant postoperative complications, such as wound infections, delayed healing, increased risk of systemic infections (sepsis), prolonged hospitalization, and the need for additional surgical interventions. Estimates suggest that SSIs contribute to an additional healthcare burden of approximately 1 billion USD annually and represent the second most common type of healthcare-associated infection [1]. Most SSIs result from the patient’s natural skin flora contaminating the surgical site during incision. However, the risk of SSIs and the associated surgical complications can be significantly reduced through an appropriate antibiotic regimen. Previously, preoperative antibiotics were administered primarily to protect high-risk patients against bacteremia-induced joint prosthesis infections or infective endocarditis. However, the rates of inappropriate antibiotic use during clean surgeries and the improper timing of delivery have increased [1]. Antibiotic misuse not only exposes patients to potential adverse effects but also contributes to the development of microbial resistance [2]. Notably, excessive antibiotic use has not been associated with a significant reduction in SSIs. One of the most common errors in modern surgical antibiotic prophylaxis is the unnecessary prolongation of antibiotic administration. Importantly, the risks associated with inappropriate antibiotic prophylaxis likely outweigh its potential benefits [3].

Given the breadth and diversity of procedures within the field of plastic surgery, maintaining adequate awareness of antibiotic prophylaxis remains challenging. Although the literature supports the use of antibiotic prophylaxis in craniofacial, breast, and cosmetic surgeries, plastic surgeons still lack comprehensive and standardized evidence-based guidelines (EBGs) to guide clinical decision-making effectively. The variability and inappropriate use of antibiotic prophylaxis may be attributed to the lack of standardized recommendations specific to plastic surgery [4]. This study aimed to evaluate the knowledge, beliefs, and practices regarding antimicrobial prophylaxis during plastic and reconstructive surgeries. It specifically explored the current pre- and postoperative antimicrobial prophylaxis practices among plastic surgeons and determined whether experiences with postoperative infections influenced deviations from established guidelines.

## Materials and methods

Study design

A questionnaire-based cross-sectional study was conducted using an online self-administered survey. The survey evaluated the current plastic surgeons’ knowledge of existing guidelines on antibiotic prophylaxis and whether the complications encountered during their practice led to modifications in their approach. The questionnaire, structured in English, was freely hosted and administered through Google Forms (Google Inc., Mountain View, Calif., USA). The survey link was distributed randomly via WhatsApp (Menlo Park, CA, USA) to all local and national Saudi plastic surgeons and their respective hospital departmental secretaries. Informed consent was obtained from all respondents prior to the study. The results of this cross-sectional study were reported in accordance with the Standards for the Reporting of Observational Studies in Epidemiology checklist. Data were collected from March 1, 2023, to May 30, 2023, after obtaining a waiver from the Institutional Review Board of King Abdullah Medical City, Makkah, Saudi Arabia. The study adhered to the ethical principles of the Declaration of Helsinki.

The sampling process did not target any specific age groups or geographical regions. Instead, the survey link was distributed to all plastic surgery consultants currently practicing in Saudi Arabia. Therefore, the inclusion criteria were met, ensuring comprehensive participation data from the eligible population. Accordingly, plastic surgery residents and non-board-certified surgeons were excluded from the study. Reminder messages were sent in weeks one, four, and six, and the questionnaire was closed at week eight.

The questionnaire comprised 13 questions, covering demographics, practice settings, antibiotic use in clean and contaminated surgeries, and knowledge and attitudes. It included four domains. The first domain assessed years of experience, practice settings, and the percentage of hand and upper [A1] limb procedures. The second domain evaluated the preoperative antibiotic use in clean surgeries, preoperative administration, antibiotic selection, postoperative prophylaxis, and the duration of postoperative treatment. The third domain examined preoperative antibiotic administration, antibiotic selection, postoperative prophylaxis, and the duration of postoperative treatment in contaminated surgeries. The last domain assessed plastic surgeons’ awareness of antibiotic guidelines and the factors influencing their prescribing decisions.

Statistical analysis

All statistical analyses were performed using IBM Corp. Released 2017. IBM SPSS Statistics for Windows, Version 25.0. Armonk, NY: IBM Corp. Descriptive statistics were used to summarize the categorical variables and presented as frequencies according to their distribution. Comparisons between groups were conducted according to the data distribution. The chi-square test was used to examine the associations between categorical variables. Statistical significance was set at a p-value of <0.05. Multivariate analysis was not feasible due to the small population size. Data imputation was necessary as the dataset was not complete for all included variables.

## Results

Our study included 65 plastic surgeons, with the vast majority (n=58, 89.2%) employed in the governmental sector, including universities, military, and National Guard hospitals, whereas only 10.8% (n=7) worked in private practice. In terms of professional experience, 35.4% of (n =23) plastic surgeons had been practicing for 10-15 years, whereas (n = 16) 24.6% had more than 20 years of experience. The percentage distribution of hand and upper limb procedures performed varied, with (n = 23) 35.4% reporting that these surgeries comprised 26%-50% of their workload, whereas only (n = 2) 3.1% performed them in 76%-100% of their practice (Table 1).

**Table 1:** Sociodemographic data (n=65).

Parameter	Frequency (n)	Percentage (%)
Work experience (years)		
0–5	7	10.8
5–10	9	13.8
10–15	23	35.4
15–20	10	15.4
>20	16	24.6
Practice settings		
Governmental (university hospitals, military hospitals, etc.)	58	89.2
Private practice hospitals	7	10.8
Percentage distribution of hand and upper limb procedures performed in practice		
0%–25%	18	27.7
26%–50%	23	35.4
51%–75%	22	33.8
76%–100%	2	3.1

Table 2 presents the participants’ knowledge and practices. Antibiotics were administered preoperatively in clean hand and upper limb surgeries by (n = 56) 86.2% of surgeons, with cefazolin being the most frequently selected antibiotic (n = 49, 75.4%). Approximately (n = 62) 95.4% reported the administration of antibiotics in clean hand and upper limb surgeries involving instrument usage (K-wire/screw), with cefazolin remaining the most commonly chosen agent (n = 50, 76.9%). For postoperative antibiotic prescriptions in clean hand surgeries, amoxicillin-clavulanate (n=31, 47.7%) was the most frequently prescribed, followed by cephalexin (n=11, 16.9%) and cefuroxime (n=10, 15.4%). Postoperative antibiotics were prescribed for more than seven days in 46.2% (n=30) of clean hand surgeries. Approximately 96.9% (n=63) of surgeons reported the administration of antibiotics in contaminated hand and upper limb surgeries, with cefazolin being the antibiotic of choice (n=50, 76.9%). The majority (n=64, 98.5%) of surgeons reported the administration of antibiotics in contaminated hand and upper limb surgeries involving instrument usage (K-wire/screw), with cefazolin being the most frequently selected antibiotic (n=48, 73.8%). Amoxicillin-clavulanate (n = 36, 55.4%) was the most frequently prescribed antibiotic for contaminated hand surgeries, followed by cefalexin (n=9, 13.8%) and clindamycin (n=3, 4.6%). Additionally, antibiotics were prescribed postoperatively for more than seven days after contaminated hand surgery (n=44, 67.7%). More than half of the participants (n=40, 61.5%) were aware of existing guidelines for antibiotic use in hand and upper limb surgeries. However, many surgeons reported using antibiotics in clean hand surgeries due to two primary factors: the lack of clear consensus or guidelines for antibiotic selection in hand and upper limb surgeries (n = 20, 30.8%) and patient expectations, which pressured them to prescribe antibiotics postoperatively (n = 20, 30.8%).

**Table 2:** Knowledge and practice of perioperative antimicrobial prophylaxis among plastic surgeons (n=65).

		Frequency (%)	Percentage (%)
In clean hand and upper limb cases, do you administer antibiotics preoperatively?	Yes, I do	56	(86.2%)
	No, I do not	9	(13.8%)
If yes, the antibiotic routinely used is:	Cefazolin	49	(75.4%)
	Cefuroxime	6	(9.2%)
	Clindamycin	1	(1.5%)
In clean hand and upper limb cases involving instrument usage (K wire/screw, etc.) do you administer antibiotics preoperatively?	Yes, I do	62	(95.4%)
	No, I do not	3 (4.6%)	(4.6%)
If yes, the antibiotic routinely used is:	Cefazolin	50	(76.9%)
	Cefuroxime	8	(12.3%)
	Cephalexin	3	(4.6%)
	Clindamycin	1	(1.5%)
If you prescribe postoperative prophylactic antibiotics in clean hand surgeries, your choice of antibiotic is:	Amoxicillin clavulanate (Augmentin)	31	(47.7%)
	Cephalexin	11	(16.9%)
	Cefuroxime	10	(15.4%)
	Cefazolin	3	(4.6%)
	Clindamycin	1	(1.5%)
	None	9	(13.8%)
The duration of the postoperative antibiotics course set for clean hand cases (days):	1-3	10	(15.4%)
	4-6	25	(38.5%)
	>7	30	(46.2%)
In contaminated hand and upper limb cases, do you administer antibiotics preoperatively?	Yes, I do	63	(96.9%)
	No, I do not	2	(3.1%)
If yes, the antibiotic routinely used is:	Cefazolin	50	(76.9%)
	Cefuroxime	9	(13.8%)
	Clindamycin	3	(4.6%)
	Vancomycin	1	(1.5%)
	Ciprofloxacin	1	(1.5%)
In contaminated hand and upper limb cases involving instrument usage (K wire/screw, etc.) do you administer antibiotics preoperatively?	Yes, I do	64	(98.5%)
	No, I do not	1	(1.5%)
If yes, the antibiotic routinely used is:	Cefazolin	48	(73.8%)
	Cefuroxime	11	(16.9%)
	Clindamycin	3	3 (4.6%)
	Vancomycin	2	2 (3.1%)
If you prescribe postoperative prophylactic antibiotics in contaminated hand surgeries, the antibiotic of choice is:	Amoxicillin clavulanate (Augmentin)	36	(55.4%)
	Cefuroxime	12	(18.5%)
	Cephalexin	9	(13.8%)
	Clindamycin	3	(4.6%)
	Cefazolin	2	(3.1%)
	None	3	(4.6%)
The duration of the postoperative antibiotic course set for contaminated hand cases (days):	1-3	2	(3.1%)
	4-6	19	(29.2%)
	>7	44	(67.7%)
Are you aware of any guidelines for antibiotics use in hand and upper limb surgery use?	Yes, I am aware	40	(61.5%)
	No, I am not aware	25	(38.5%)
Which of the following is a major motive behind choosing to administer and/or prescribe antibiotics in clean hand cases?	There is no clear consensus or guideline for antibiotic choice in hand and upper limb surgeries	20	(30.8%)
	Patients are expecting to have antibiotics postoperatively therefore; I am pressured to administer them	20	(30.8%)
	I am afraid of legal claims if the patient developed an infection if I did not prescribe antibiotics	15	(23.1%)
	I do not trust the sterility of the field and the aseptic techniques performed	10	(15.3%)

Table 3 investigates the association between workplace environment and knowledge/practice variables related to surgical antimicrobial prophylaxis. No statistically significant differences were observed between governmental and private sector surgeons across all comparisons (p > 0.05). Additionally, all chi-square values were low (χ² range: 0.001-0.385), with corresponding degrees of freedom = 1 and negligible effect sizes (Cramér’s V = 0.000-0.077), indicating no meaningful association.

**Table 3:** The association between workplace environment and knowledge.

		Environment		Chi-square χ² (df = 1)	P-value
		Governmental	Private sector		
In clean hand and upper limb cases, do you administer antibiotics preoperatively?	Yes, I do	50 (89.3%)	6 (10.7%)	0.001	0.972
	No, I do not	8 (88.9%)	1 (11.1%)		
In clean hand and upper limb cases involving instrument usage, do you administer antibiotics preoperatively?	Yes, I do	55 (88.7%)	7 (11.3%)	0.385	0.538
	No, I do not	3 (100%)	0		
In contaminated hand and upper limb cases, do you administer antibiotics preoperatively?	Yes, I do	56 (88.9%)	7 (11.1%)	0.247	0.618
	No, I do not	2 (100%)	0		
In contaminated hand and upper limb cases involving instrument usage do you administer antibiotics preoperatively?	Yes, I do	57 (89.1%)	7 (10.9%)	0.121	0.726
	No, I do not	1 (100%)	0		
Are you aware of any guidelines for antibiotics use in hand and upper limb surgery use?	Yes, I am aware	36 (90%)	4 (10%)	0.064	0.8
	No, I am not aware	22 (88%)	3 (12%)		

## Discussion

No comprehensive clinical practice guidelines exist regarding antibiotic prophylaxis in upper limb surgery. One factor leading to national trends in antimicrobial resistance is the inappropriate use of SAP [5]. Several factors contribute to the misuse of SAP, with three primary categories emerging from the plastic surgeons’ perspectives in the current study.

In this study, antibiotics were administered preoperatively by most surgeons to their patients in both clean and contaminated hand and upper limb surgeries (86.2% and 96.9%, respectively), with a slightly higher prevalence in contaminated surgeries. The use of antibiotics was also reported in the majority of clean and contaminated surgeries involving instrument usage (such as K-wires or screws). SAP is routinely administered to patients undergoing clean or clean-contaminated procedures to prevent SSIs before, during, or after surgery [6]. According to the Centers for Disease Control, a clean wound is defined as an incision through which the respiratory, alimentary, or genitourinary tracts are not entered during a surgical procedure, with no evidence of inflammation and no breach in the sterile technique [6]. By contrast, a clean-contaminated wound is an incision through which the respiratory, alimentary, or genitourinary tracts are entered under controlled conditions without contamination. However, recent evidence has questioned the necessity of SAP in clean elective hand surgeries. A systematic review by Negri et al. found no significant reduction in SSI risk with prophylactic antibiotics in clean, soft tissue procedures of the upper limb [7]. Likewise, Tosti et al. demonstrated no difference in infection rates in clean cases with or without prophylaxis [8]. 

Cefazolin was the most frequently prescribed antibiotic. Similarly, Yu reported that more than half of the participants preferred cefazolin [9]. As a first-generation cephalosporin, cefazolin is recommended in various guidelines for its broad antibacterial coverage across multiple bodily systems [10]. However, it cannot pass through the blood-brain barrier. Cefazolin exhibits primary activity against *Staphylococcus aureus* and other cutaneous bacteria, reaching peak activity following intravenous injection and within 10 min of intramuscular administration. Its minimum inhibitory concentration is maintained for an adequate duration due to its half-life, which ranges from 90 to 150 min [11]. Furthermore, cefazolin remains a commonly utilized agent for surgical prophylaxis in extremity surgeries, including upper limb procedures, due to its efficacy and favorable pharmacokinetic profile [12].

Postoperatively, amoxicillin-clavulanate was the most frequently prescribed antibiotic for both clean (47.7%) and contaminated (55.4%) hand surgeries. In most participants, postoperative antibiotics were prescribed for more than seven days following clean hand surgeries. According to a survey by Klifto et al., most surgeons routinely administered prophylactic antibiotics. However, many did not strictly adhere to guidelines regarding timing and duration. For example, antibiotics were often administered either earlier or later than recommended or continued for longer than necessary after surgery. This noncompliance with established guidelines may lead to suboptimal outcomes, including the development of antibiotic resistance and an increased risk of SSIs [13]. Moreover, Rafati et al. found that in 40.2% of surgical cases, the duration of antibiotic prophylaxis exceeded the recommended guidelines, highlighting the need for improved adherence to previously established protocols in order to prevent antibiotic resistance and reduce the risk of surgical site infections [14]. 

More than half of the participants in this study (61.5%) were aware of the guidelines for antibiotic use during hand and upper limb surgeries. By contrast, Mmari reported three key barriers to adherence: poorly understood hospital guidelines, a lack of hospital data on SSI rates, and the absence of hospital data on bacterial resistance patterns [15]. A prolonged course of SAP was often perceived as beneficial to the patient, whereas surgeons found reassurance in reducing the perceived risk of infection through their discretion. According to a systematic review by Ng et al., decision-making regarding SAP use is influenced by initial training and the lack of understanding of the guidelines governing its use [16]. Several factors, including personal preferences and peer pressure, further hinder guideline adherence. This is echoed by Seidelman et al., who identified both knowledge gaps and institutional inertia as persistent contributors to non-adherence in surgical prophylaxis [17].

More than half of the surgeons reported using antibiotics in clean hand surgeries for two primary reasons: the absence of a clear consensus or guidelines on antibiotic selection for hand and upper limb surgeries (30.8%) and perceived patient expectations for postoperative antibiotic administration, which created pressure to prescribe them (30.8%). These sentiments are consistent with findings by Surendran et al., who reported that patient satisfaction scores and legal concerns often influence the overprescription of antibiotics, even in clinically unjustified cases [18].

Plastic surgeons should prioritize key strategies to improve their understanding and application of perioperative antimicrobial prophylaxis. To ensure that surgeons remain current with best practices, improvements in education and training are essential. Specifically, the AMP guidelines should be incorporated into the surgical curricula and continuing medical education programs. Additionally, professional organizations, such as the American Society of Plastic Surgeons, should focus on developing and widely disseminating clear, evidence-based guidelines that are both practical and applicable in clinical settings. McKay et al. found that educational interventions, audit feedback, and clinical reminders significantly improved SAP compliance in upper extremity surgery [19].

When combined with feedback systems, routine audits of antimicrobial prophylaxis procedures can help identify practice gaps and promote policy adherence. Hospitals must align their protocols with the latest EBGs and use electronic medical records to support surgeons in the effective implementation of antimicrobial prophylaxis. Therefore, institutional support is crucial. Finally, ongoing research and continuous monitoring of antimicrobial prophylaxis practices and outcomes are required to improve patient care, refine recommendations, and ultimately reduce the incidence of SSIs. Studies, such as the one by Gouvêa et al., have assessed the variability in adherence to surgical antibiotic prophylaxis guidelines across various surgical specialties, including plastic surgery. Their findings clearly highlighted the importance of implementing antimicrobial stewardship programs to standardize current practices and improve compliance with established guidelines [20].

Limitations

Our study faced several limitations that must be addressed. Due to the nature of our study design, our self-reported survey is subject to respondent recall and interpretation biases; moreover, due to it being a self-administered survey, our results may be biased. Furthermore, the results may be affected due to non-response bias. Lastly, as the authors do not know the actual number of active plastic surgery consultants in the country, there is no definitive way to confirm the total number. We worked diligently to include as many responses as possible in order to reflect the current practice, which we believe to have achieved in this study.

## Conclusions

Most plastic surgeons adhered to the use of pre- and postoperative SAP in both contaminated and clean hand surgeries. Although the significance of antimicrobial prophylaxis is widely recognized, inconsistencies in its practical application remain a challenge. Future studies should aim at enhancing adherence to best current practices in order to reduce the risk of SSIs and improve patient outcomes, as they require a multifaceted approach that includes education, clear and standardized guidelines, regular audits, and strong institutional support.
